# Trajectories of pain and depressive symptoms among people living with low back pain during the COVID-19 pandemic: a 24-month longitudinal study

**DOI:** 10.1097/PR9.0000000000001165

**Published:** 2024-05-31

**Authors:** Adriana Angarita-Fonseca, Mathieu Roy, Anaïs Lacasse, Guillaume Léonard, Pierre Rainville, Marie-France Marin, Iulia Tufa, Erika L. Gentile, M. Gabrielle Pagé

**Affiliations:** a Department of Health Sciences, Université du Québec en Abitibi-Témiscamingue (UQAT), Rouyn-Noranda, QC, Canada; b Research Center of the Centre hospitalier de l’Université de Montréal, Montreal, QC, Canada; c Department of Psychology, McGill University, Montreal, QC, Canada; d Alan Edwards Centre for Research on Pain, McGill University, Montreal, QC, Canada; e Research Center on Aging, CIUSSS de l’Estrie-CHUS; School of Rehabilitation, Faculty of Medicine and Health Sciences, Université de Sherbrooke, Sherbrooke, QC, Canada; f Department of Stomatology, Faculty of Dentistry, Université de Montréal, Montreal, QC, Canada; g Centre de recherche de l'Institut universitaire de gériatrie de Montréal (CRIUGM), Montreal, QC, Canada; h Department of Psychology, Université de Québec à Montréal, Montreal, QC, Canada; i McGill University Health Center, Montreal, QC, Canada; j Quebec Pain Research Network, QC, Canada; k Department of Anesthesiology and Pain Medicine, Université de Montréal, Montreal, QC, Canada

**Keywords:** Low back pain, COVID-19, Cortisol, Trajectories, Depression

## Abstract

**Introduction:** We explored trajectories of pain intensity and depressive symptoms over the first 24 months of the pandemic in people with low back pain.

**Methods:** This longitudinal study was conducted alongside the Quebec Low Back Pain Study. Starting in April 2020 and every 3 months until July 2022, 291 participants completed an online survey. Group-based trajectory modeling was used to identify patterns of pain intensity and depressive symptoms. Onset outbreak characteristics were then put in relation with trajectory groups using multivariate logistic regression.

**Results:** The analysis revealed 5 trajectories of pain intensity and depressive symptoms, respectively. The pain trajectories were stable mild (n = 17, 5.8%); stable moderate (n = 103, 35.4%); stable severe (n = 81, 27.8%); U-shape (n = 24, 8.3%), and inverted U-shape (n = 66, 22.7%). The trajectories of depressive symptoms were stable none (n = 58, 19.9%); stable very mild (n = 61, 21.0%); stable mild (n = 85, 29.2%); stable moderate (n = 59, 21.7%); and severe slightly improving (n = 24, 8.3%). Pre-COVID everyday/nearly everyday pain, average pain intensity, and widespread bodily pain were predictive of pain trajectory groups. Higher pre-COVID depression, acute stress disorder, and lockdown measures-related stress were associated with moderate/severe depressive trajectories.

**Discussion:** Our findings indicated relative stability of pain and depressive symptoms among participants during the COVID-19 pandemic but also highlighted subgroups of people who experienced temporary deterioration or improvement over the first months of the pandemic that then reverted back to baseline levels. Modifiable risk factors were identified before the onset of the pandemic, which could give preventive measures in targeted populations.

## Abstract 2

During the first 2 years of the COVID-19 pandemic, subgroups of people experience deterioration/improvement in pain and depressive symptoms but that revert back to baseline.

## 1. Introduction

The coronavirus disease-2019 (COVID-19) pandemic and its health, social, and economic consequences initially led to a global impact on mental health compared with pre–COVID-19 levels.^37^ Lockdowns and restrictions have affected people's lives, including limited social contact, increased global stress, decreased physical activity levels, and negative mental health effects.^3^ These effects could be amplified in vulnerable populations such as people with chronic low back pain (LBP).^3^

Studies investigating the worsening of pain severity during COVID-19 among people living with chronic pain were mainly cross-sectional and focused on perceptions of change in pain symptoms.^38^ Other studies retrospectively reported pre–COVID-19 pain severity.^11,33^ These studies are limited by recall biases.^14,39,40^ Moreover, a systematic review showed a rise in the prevalence and intensity of LBP related to COVID-19.^34^

Only a few longitudinal studies have considered pain characteristics among people with LBP during the pandemic. Studies comparing prepandemic and postpandemic scores point toward the presence of subgroups, with some individuals showing improved pain scores, whereas others showing pain deterioration.^1,10^ Similarly, unchanged pain, emotional distress, and opioid misuse patterns were found over a 1-year period among adults with chronic pain,^27^ whereas improvements in pain intensity and depression scores were found in patients with chronic pain when studied over the course of the first 2 years of the pandemic.^47^ Such understanding of individuals' long-term pain and psychological distress trajectories taking into account prepandemic status, however, is lacking. It is possible that individual's stress response plays a role in the observed interindividual variability in pain trajectories.^9,31,43^

Surprisingly, although the prevalence of psychological distress among general population during the COVID-19 pandemic has been well covered, biological variables such as cortisol have been less studied.^45^ Cortisol is a hormone produced by the adrenal glands in response to stress, and it plays a vital role in regulating various physiological processes.^46^ In general, high levels of cortisol have been associated with increased pain sensitivity and exacerbation of chronic pain conditions.^18^ Elevated cortisol levels in response to stress have also been associated with major depressive disorder.^29^

Further research, particularly when pre–COVID-19 data are available, is crucial to understand the longitudinal evolution of onset outbreak and post–COVID-19 pain-related outcomes as well as identifying subgroups of patients with LBP who share similar patterns. Well-designed studies incorporating trajectory analysis can help elucidate how the COVID-19 pandemic has influenced long-term pain outcomes among people living with LBP and their associated factors. The findings of such research can inform clinical practice, public health interventions, and strategies for managing LBP in the aftermath of the pandemic. Hence, this study aimed to (1) identify trajectories of pain intensity and depressive symptoms and (2) determine whether prepandemic and onset outbreak variables (including biological measurements of stress) are associated with trajectory groups.

## 2. Methodology

STROBE reporting guidelines were followed.

### 2.1. Recruitment and procedures

This cohort study was conducted using data from the Quebec LBP Study (QLBPS).^32^ This cohort began recruiting participants with LBP in 11/2018 (clinicaltrials.gov: NCT04791891) through mostly social and mass media, individual and institutions by email list servers, emails to patient organizations and professional societies, unions of workers at risk of LBP, and health care clinics. Participants completed online questionnaires over a 24-month period. In March 2020, 1 month after the declaration of the state of emergency in Quebec, Canada, all active QLBPS cohort participants were invited to participate in a special COVID-19 study, which required them to complete specific questionnaires every 3 months until July 2022 and, if interested, provide hair sample 3 months after the start of the pandemic (June 2020). For this study, eligible participants were adults (≥18 years old) fluent in French, who had LBP for more than 3 months, were active participants in the QLBPS, and completed at least 2 time points of the COVID-19 study questionnaires.

### 2.2. Measures

#### 2.2.1. Outcomes

Pain intensity was evaluated using the Numeric Rating Scale (NRS-11) from the Canadian Minimum Dataset for Chronic LBP Research, as follows: “In the past 7 days, how would you rate your average lower back pain?”.^23^ Possible answers range from 0 (no pain) to 10 (worst imaginable pain). Depressive symptoms were evaluated using the Emotional Distress—Depression PROMIS Short-Form 4a,^21^ which had 4 questions (In the past 7 days, (a) I felt hopeless, (b) I felt helpless, (c) I felt depressed, and (d) I felt hopeless) on a 5-point Likert scale (Cronbach alpha >0.90^19^). The raw score was computed summing the 4 items and then rescaled into a T-score as per recommendations about this scale.^36^

#### 2.2.2. Potential predictors

The predictors of trajectory groups included pre–COVID-19 and onset outbreak COVID-19–related variables.

The pre–COVID-19 variables were taken from the latest evaluation (between 0 and 17 months) from the QLBPS (nearest to the COVID-19 study time 0), which was based on the Canadian Minimum Dataset for Chronic LBP Research.^2,13,23^ This included the following characteristics: LBP status and type; comorbid painful conditions; history of LBP surgical interventions; pain interference (PROMIS Short-Form 4a^36^); LBP treatments (opioid use, infiltrations/injections, exercise therapy, and psychological counselling); LBP-related workplace absenteeism and compensation benefits; physical function (PROMIS Short-Form 4a^36^); kinesiophobia (an item from the STarT Back Screening Tool^5^); catastrophizing (an item from the STarT Back Screening Tool^5^); LBP-related lawsuits and legal claims; substance abuse; sociodemographic profile (age, sex identity, indigenous membership, racialized groups membership, employment, and education level); cigarette use; and height/weight to calculate body mass index. Sex at birth and administrative region were also available in the QLBPS. Internal consistency for the multi-item measures of the PROMIS is excellent (Cronbach alpha >0.90^19^).

Onset outbreak COVID-19 pandemic variables were selected by a group of pain researchers, clinicians, and persons with lived experience involved in the QLBPS who developed a questionnaire to measure stressors related to the onset of the pandemic. Acute stress disorder symptoms (19-item Acute Stress Disorder Scale) were collected by asking the extent to which they experienced symptoms of acute stress such as hypervigilance, numbing, arousal, and reactivity when they thought or heard about COVID-19 on a scale from 0 (not at all) to 4 (extremely) (Cronbach alpha >0.90).^8^ Participants also reported their level of stress by answering the following questions developed for the purpose of this study: “To what extent do you find the COVID-19 pandemic stressful?” and “To what extent do you find the lockdown measures associated with the COVID-19 pandemic stressful?” with possible answers ranging from 0 (not at all) to 10 (extremely).^33^ In addition, samples measuring 6 centimeters of hair were collected between June 2020 and July 2020 by mail to assess cortisol levels (stress hormone). Samples were taken by the participants themselves with instructions and material sent to them by mail. As hair grows an estimated 1 cm/month, the centimeters closest to the scalp represent the previous month.^17^ By collecting 6 cm of hair sample, it was possible to produce an estimate of cortisol levels on average in the first 3 months of the pandemic (first 3 cm of hair closest to the scalp—March 2020 to June 2020) and in the 3 months before the onset of the pandemic (next 3 cm of hair—December 2019 to March 2020) to obtain percentage of changes in cortisol levels. Hair samples provide a cumulative measure of cortisol levels, thus eliminating the drastic variations by timing of sample collection using saliva, urine, or serum.^24^ The hair samples were stored at room temperature in a dark and dry environment. The samples were centrally assayed to determine hair cortisol. Information regarding COVID-19 diagnosis during each evaluation was collected by inquiring, “Have you been diagnosed with COVID-19 (confirmed by a test that was administered by a health care professional)?” Finally, given that the pre–COVID-19 questionnaire was not filled at the same time by all participants (we took their latest QLPBS survey completed from this cohort before the pandemic), a time variable representing the number of months elapsed between the pre–COVID-19 QLPBS survey and the pandemic onset was calculated.

### 2.3. Statistical analysis

The pain intensity and depression scores collected from time 0 (April 2020) to time 9 (July 2022) were modelled into trajectories using group-based trajectory modelling (GBTM).^28^ Trajectory groups were then used as categorical dependent variables.

The GBTM determines the form and number of groups that best fit the data and provides a metric for evaluating the precision of group assignments.^30^ It tolerates missing data well.^30^ Up to 5 trajectories (groups) and 3 trajectory shapes (linear, quadratic, and cubic) were tested. To select the right number of trajectories, several censored normal (CNORM) models were evaluated. We evaluated up to 5 distinct trajectories and 3 trajectory shapes: (1) linear (straight line), (2) quadratic (U-shaped curve/parabola), and (3) cubic. Each trajectory within the same model could have its own shape. Therefore, there were 3 possible combinations of trajectory shapes for models including only 1 trajectory (eg, 1, 2, or 3), 9 models with 2 trajectories (eg, 11, 22, 33, 12, 13, 23, 21, 31, and 32), 27 models with 3 trajectories (eg, 112, 221, and 333), 81 models with 4 trajectories (eg, 1121, 3221, and 3333), and 243 models with 5 trajectories (eg, order: 11211, 32211, and 33333). In total, GBTM led to the testing of 363 models. For pain and depression, respectively, the models with the lower value (absolute value) of the Bayesian information criterion (BIC), with posterior probabilities >0.70, and with all trajectories being represented by at least 5% of participants were selected.^28^ The trajectory group profiles were compared using medians and percentages and the 95% confidence interval for multinomial proportions and medians.

Multiple logistic regression models using LASSO with *k*-fold cross-validation (*k* = 10) were then applied across the study sample to assess the association between prepandemic and onset outbreak variables (independent variables) and dichotomized pain intensity trajectory groups (dependent variables) while accounting for covariables. In the 4 regression models, pain intensity trajectory groups were dichotomized as follows: (1) stable severe and moderate pain intensity vs other trajectory groups, (2) U-shape pain intensity vs other trajectory groups, (3) inverted U-shape pain intensity vs other trajectory groups, and (4) stable mild pain intensity vs other trajectory groups. As for depressive symptoms trajectory groups, 3 regression models were compared: (1) severe slightly improving and stable moderate depressive symptoms vs other trajectory groups, (2) stable mild depressive symptoms vs other trajectory groups, and (3) stable none and very mild depressive symptoms vs other trajectory groups. Although a broad set of a priori determined variables potentially associated with changes in pain intensity and depressive symptoms were included in the bivariate logistic regression models, only the most valuable variables according to the Least Absolute Shrinkage and Selection Operator (LASSO^41^) regression were included in the final model. Age, sex, acute stress disorder symptoms, how stressful the COVID-19 pandemic was perceived, how stressful the lockdown measures associated with the COVID-19 pandemic were perceived, and having received a COVID-19 positive screening were forced in the models. Multicollinearity was tested according to variance inflation factors.^44^

Finally, the comparison of the pre-onset of the COVID-19 pandemic and post-onset of the COVID-19 pandemic percentage of changes in cortisol levels (pg/mg) among the trajectory groups was conducted using medians and their corresponding 95% confidence intervals in a subset of participants. Multiple logistic regression models, which included the pre-onset of the COVID-19 pandemic and post-onset of the COVID-19 pandemic percentage changes in cortisol levels, were adjusted for various factors. These factors encompassed age in years, pre-onset of the COVID-19 pandemic pain intensity, pre-onset of the COVID-19 pandemic depressive symptoms, pre-onset of the COVID-19 pandemic pain interference, pre-onset of the COVID-19 pandemic physical function, scores indicating the perception of the COVID-19 pandemic as stressful, and the perception of lockdown measures associated with the COVID-19 pandemic as stressful during the early pandemic stages, as well as the duration in months between the pre-onset of the COVID-19 pandemic and onset-of-pandemic surveys. All analyses were performed using SAS (version 9.4, Cary, NC).

## 3. Results

**Figure 1.**
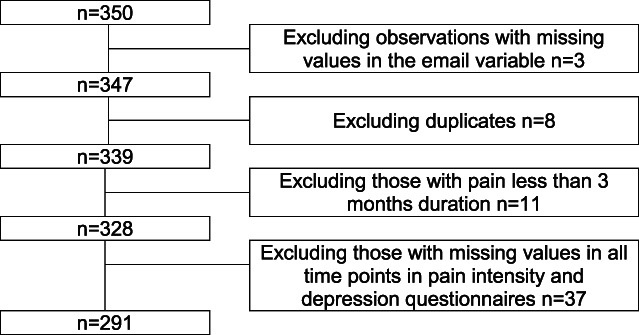
Flowchart of the study.

The analysis was conducted among 291 participants living with LBP for at least 3 months (Fig. 1). Percentage of missing data ranged from 0.7% at baseline to 55% at 24-month follow-up, with an average of 36% of missing data per time point. The sample characteristics are shown in Table 1. Mean age at the onset of pandemic was 47.4 ± 12.3 years, and 69.4% were female.

**Table 1:** Sample characteristics.

Variables	Category	Frequency	Percent
Pre–COVID-19 variables			
Sex at birth	Missing Female Male	3 202 86	1.0 69.4 29.6
Working (full time or part-time)	No Yes	257 34	88.3 11.7
Low back pain been an ongoing problem over the past 6 mo	Every day or nearly every day in the past 6 mo At least half the days in the past 6 months Less than half the days in the past 6 mo	132 85 74	45.4 29.2 25.4
Low back pain spread down your leg(s) during the past 2 wk	No/not sure Yes	131 160	45.0 55.0
Widespread pain (pain in most of the body)	Missing No Yes	7 129 155	2.4 44.3 53.3
Opioid painkillers	No/not sure Yes	220 71	75.6 24.4
Infiltrations/injections	Missing No/not sure Yes	2 253 36	0.7 86.9 12.4
It is not really safe for a person with my low back problem to be physically active	Agree Disagree	57 234	19.6 80.4
Feeling that my low back pain is terrible and never going to get any better	Agree Disagree	125 166	43.0 57.0
Excess of alcohol or drugs	Missing No Yes	1 105 185	0.3 36.1 63.6
Current smoker	Missing No Yes	10 234 47	3.4 80.4 16.2
Body mass index ≥30	Missing <30 ≥30	7 167 117	2.4 57.4 40.2
	Score, n	Mean ± SD	Median (IQR)
Pain intensity before COVID-19	Score from 0 = no pain to 10 = worst imaginable pain) n = 291	5.9 ± 1.9	6.0 (5.0, 7.0)
Depression score before COVID-19	T score from 41.0 to 79.4, n = 287	57.2 ± 9.4	58.9 (51.8, 63.9)
Pain interference before COVID-19	T score from 41.6 to 75.6, n = 286	61.8 ± 6.7	62.5 (57.1, 66.6)
Physical function before COVID-19	T score from 22.9 to 56.9, n = 286	41.0 ± 6.5	40.4 (36.7, 45.3)
Onset-of-pandemic variables	Category	Frequency	Percent
Age groups (y)	Missing ≤50 >50	10 172 109	3.4 59.1 37.5
	Score range, n	Mean ± SD	Median (IQR)
Acute stress disorder	Score from 0 to 76, n = 291	17.0 ± 13.4	14.0 (7.0, 23.0)
Finding the COVID-19 pandemic stressful	Score from 0 (not at all) to 10 (extremely), n = 290	6.8 ± 2.5	7.0 (5.0, 9.0)
Finding lockdown measures associated with the COVID-19 pandemic stressful	Score from 0 (not at all) to 10 (extremely), n = 291	5.6 ± 2.8	6.0 (4.0, 8.0)
Cortisol variables	Score range, n	Mean ± SD	Median (IQR)
Cortisol before COVID-19	pg/mg, n = 89	34.6 ± 52.6	16.3 (10.1, 33.9)
Cortisol after COVID-19	pg/mg, n = 74	30.1 ± 70.9	11.8 (7.5, 21.0)
Percentage of change in cortisol	pg/mg, n = 74	37.5 ± 55.9	24.5 (−3.0, 58.3)
Time-variant variable	Category	Frequency	Percent
COVID-19 diagnosis at any point	No Yes	245 46	84.2 15.8
Methodological variable	Score range, n	Mean ± SD	Median (IQR)
Months between pre–COVID-19 and onset-of-pandemic surveys	0–17 mo, n = 289	5.2 ± 4.2	4.0 (1.0, 9.0)

IQR, interquartile range.

The best fit for the data keeping at least 5% of participants in the smallest group trajectory was a five-trajectory model for pain (23321) and a five-trajectory model for depressive symptoms (11111). Model fit indices for the best 15 models tested are shown in Appendix A and B, http://links.lww.com/PR9/A234.

**Figure 2.**
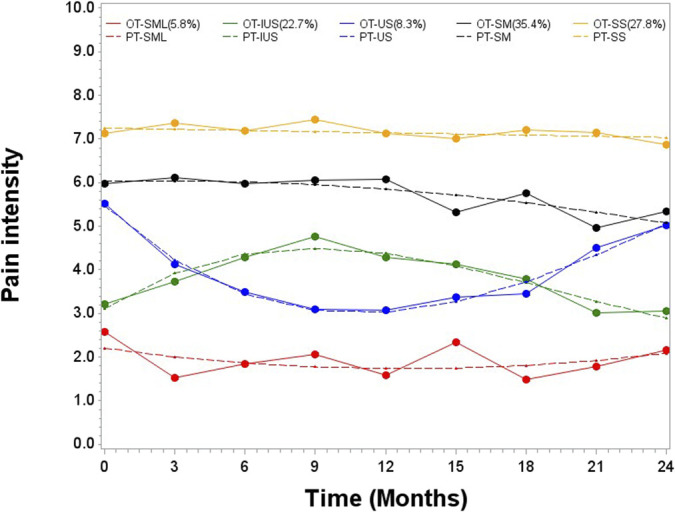
Observed (OT) and predicted (PT) trajectories of pain intensity. IUS, inverted U-shape; S, stable severe; SM, stable moderate; SS, stable slight; US, U-shape.

The trajectories of pain intensity were (1) stable mild (n = 17, 5.8%, median score = 1.5 (IQR = 1.0, 3.0)); (2) stable moderate (n = 103, 35.4%, median score = 5.0 (IQR = 4.0, 7.0)); stable severe (n = 81, 27.8%, median score = 7.0 (IQR = 7.0, 8.0)); U-shape (n = 24, 8.3%, median score = 4.0 (IQR = 2.0, 5.0)); and inverted U-shape (n = 66, 22.7%, median score = 4.0 (IQR = 3.0, 5.0)). Pain intensity trajectories can be found in Figure 2. Group-based trajectory modelling parameter coefficients are available in Appendix C, http://links.lww.com/PR9/A234 and pain intensity scores by trajectory groups in Appendix D, http://links.lww.com/PR9/A234.

**Figure 3.**
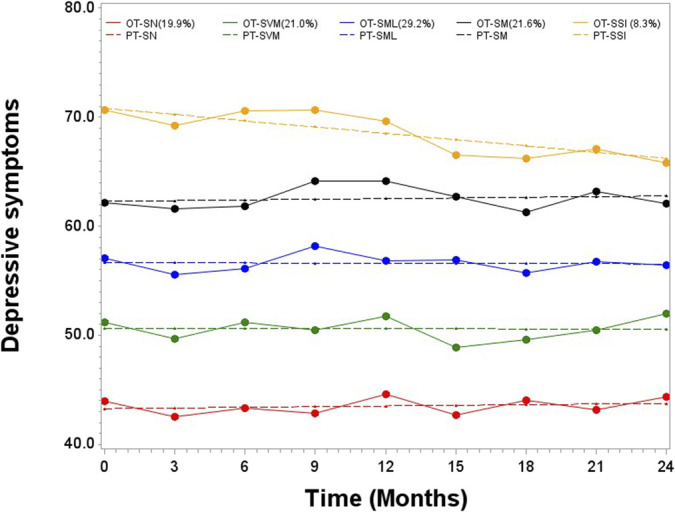
Observed (OT) and predicted (PT) trajectories of depressive symptoms. S, severe slightly improving; SM, stable moderate; SML, stable mild; SN, stable none; SS, stable very mild.

The trajectories of depressive symptoms were (1) stable none (n = 58, 19.9%, median score 41.0 (IQR = 41.0, 41.0)); (2) stable very mild (n = 61, 21,0%, median score = 51.8 (IQR = 49.0,53.9)); (3) stable mild (n = 85, 29,2%, median score = 57.3 (IQR = 53.9, 58.9)); (4) stable moderate (n = 63, 21.6%, median score = 62.2 (IQR = 58.9, 65.7)); and (5) severe slightly improving (n = 24, 8.3%, median score = 69.4 (IQR = 67.5, 71.2)). The depressive symptoms trajectories can be found in Figure 3. Group-based trajectory modelling parameter coefficients are available in Appendix E, http://links.lww.com/PR9/A234 and depressive symptoms scores by trajectory groups in Appendix F, http://links.lww.com/PR9/A234.

Descriptively, the stable mild pain intensity trajectory was characterized by the absence of participants working or current smokers, a low percentage of widespread pain and catastrophic thinking, and low average pain intensity before COVID-19. The severe pain trajectory was characterized by a high percentage of people reporting widespread pain and catastrophizing thinking, as well as high average pain intensity, depressive symptoms, and high levels of pain interference before COVID-19 (Table 2), and a low percentage of people reporting pain less than half the days in the past 6 months. In the multivariate logistic regression (Table 3), the stable severe and stable moderate pain intensity trajectories were associated with having pain every day or nearly every day in the past 6 months (OR = 3.3; 95% CI = 1.4, 7.7), widespread pain (OR = 2.7; 95% CI = 4.0, 5.2), and higher pain intensity before COVID-19 (OR = 1.5; 95% CI = 1.3, 1.9) compared with the other 3 trajectories. The U-shape trajectory was associated with less pain interference before COVID-19 (OR = 0.9; 95% CI = 0.8, 0.98) and having COVID-19 at any point (OR = 3.9; 95% CI = 1.3, 11.3) compared with the other 4 trajectories. The inverted U-shape was associated with lower pain intensity before COVID-19 (OR = 0.8; 95% CI = 0.7, 0.9) compared with the other 4 trajectories. The stable slight pain trajectory was associated with pain that radiates down the leg (OR = 0.1; 95% CI = 0.1, 0.7) and widespread pain (OR = 0.1; 95% CI = 0.01, 0.6) compared with the other 4 trajectories. With the exception of having COVID-19 at any point, none of the factors associated with the pandemic were associated with trajectory groups.

**Table 2:** Sample characteristics by trajectories of pain intensity.

Variable	Category	Stable mild		Inverted U-shape		U-shape		Stable moderate		Stable severe	
		n	% (95% CI)	n	% (95% CI)	n	% (95% CI)	n	% (95% CI)	n	% (95% CI)
Pre–COVID-19 variables											
Sex at birth	Female	11	64.7 (38.3, 84.4)	51	77.3 (64.0, 86.7)	20	83.3 (61.0, 94.1)	61	59.2 (47.4, 70.0)	59	72.8 (60.7, 82.3)
	Male	6	35.3 (15.6, 61.7)	15	22.7 (13.3, 36.0)	4	16.7 (5.9, 39.0)	39	37.9 (27.3, 49.7)	22	27.2 (17.7, 39.3)
Working (full time or part-time)	No	17	100.0 (81.6, 100.0)	58	87.9 (76.1, 94.3)	20	83.3 (61.0, 94.1)	90	87.4 (78.3, 93.0)	72	88.9 (78.7, 94.5)
	Yes	0	0	8	12.1 (5.7, 23.9)	4	16.7 (5.9, 39.0)	13	12.6 (7.0, 21.7)	9	11.1 (5.5, 21.3)
Low back pain been an ongoing problem over the past 6 mo	At least half the days in the past 6 mo	2	11.8 (2.6, 40.2)	21	31.8 (20.0, 46.5)	10	41.7 (21.6, 65.0)	32	31.1 (21.4, 42.7)	20	24.7 (15.2, 37.6)
	Every day or nearly every day in the past 6 mo	5	29.4 (11.1, 58.1)	17	25.8 (15.2, 40.2)	4	16.7 (5.5, 40.7)	52	50.5 (39.0, 61.9)	54	66.7 (53.4, 77.7)
	Less than half the days in the past 6 mo	10	58.8 (31.8, 81.4)	28	42.4 (29.0, 57.0)	10	41.7 (21.6, 65.0)	19	18.4 (11.1, 29.2)	7	8.6 (3.7, 19.1)
Low back pain spread down your leg(s) during the past 2 wk	No/not sure	15	88.2 (61.8, 97.2)	35	53.0 (39.5, 66.1)	15	62.5 (40.1, 80.6)	39	37.9 (28.0, 48.9)	27	33.3 (22.9, 45.7)
	Yes	2	11.8 (2.8, 38.2)	31	47.0 (33.9, 60.5)	9	37.5 (19.4, 59.9)	64	62.1 (51.1, 72.0)	54	66.7 (54.3, 77.1)
Widespread pain (pain in most of the body)	No	15	88.2 (61.8, 97.2)	41	62.1 (48.3, 74.2)	15	62.5 (38.7, 81.5)	37	35.9 (25.6, 47.7)	21	25.9 (16.1, 38.9)
	Yes	2	11.8 (2.8, 38.2)	25	37.9 (25.8, 51.7)	8	33.3 (15.6, 57.5)	63	61.2 (49.4, 71.8)	57	70.4 (57.2, 80.8)
Opioid painkillers	No/not sure	15	88.2 (61.8, 97.2)	60	90.9 (79.8, 96.2)	20	83.3 (61.0, 94.1)	72	69.9 (59.0, 78.9)	53	65.4 (53.0, 76.1)
	Yes	2	11.8 (2.8, 38.2)	6	9.1 (3.8, 20.2)	4	16.7 (5.9, 39.0)	31	30.1 (21.1, 41.0)	28	34.6 (23.9, 47.0)
Infiltrations/injections	No/not sure	16	94.1 (69.0, 99.1)	61	92.4 (81.8, 97.1)	24	100.0 (86.2, 100.0)	86	83.5 (73.0, 90.4)	66	81.5 (69.2, 89.6)
	Yes	1	5.9 (0.9, 31.0)	5	7.6 (2.9, 18.2)		0	16	15.5 (8.8, 25.9)	14	17.3 (9.5, 29.4)
It is not really safe for a person with my low back problem to be physically active	Agree	2	11.8 (2.8, 38.2)	8	12.1 (5.7, 23.9)	3	12.5 (3.8, 34.2)	19	18.4 (11.4, 28.4)	25	30.9 (20.8, 43.2)
	Disagree	15	88.2 (61.8, 97.2)	58	87.9 (76.1, 94.3)	21	87.5 (65.8, 96.2)	84	81.6 (71.6, 88.6)	56	69.1 (56.8, 79.2)
Feeling that my low back pain is terrible and never going to get any better	Agree	1	5.9 (0.9, 31.0)	18	27.3 (16.9, 40.8)	5	20.8 (8.2, 43.5)	51	49.5 (38.8, 60.3)	50	61.7 (49.3, 72.8)
	Disagree	16	94.1 (69.0, 99.1)	48	72.7 (59.2, 83.1)	19	79.2 (56.5, 91.8)	52	50.5 (39.7, 61.2)	31	38.3 (27.2, 50.7)
Excess of alcohol or drugs	No	5	29.4 (11.8, 56.4)	24	36.4 (24.5, 50.2)	8	33.3 (15.6, 57.5)	39	37.9 (28.0, 48.9)	29	35.8 (25.0, 48.2)
	Yes	12	70.6 (43.6, 88.2)	42	63.6 (49.8, 75.5)	15	62.5 (38.7, 81.5)	64	62.1 (51.1, 72.0)	52	64.2 (51.8, 75.0)
Current smoker	No	17	100.0 (81.6, 100.0)	58	87.9 (75.1, 94.6)	22	91.7 (70.9, 98.0)	82	79.6 (68.7, 87.4)	55	67.9 (54.7, 78.8)
	Yes	0	0	7	10.6 (4.5, 23.0)	2	8.3 (2.0, 29.1)	17	16.5 (9.6, 27.0)	21	25.9 (16.1, 38.9)
Body mass index ≥30	<30	14	82.4 (55.3, 94.6)	41	62.1 (47.4, 74.9)	12	50.0 (29.2, 70.8)	61	59.2 (47.4, 70.0)	39	48.1 (35.4, 61.1)
	≥30	3	17.6 (5.4, 44.7)	24	36.4 (23.8, 51.1)	12	50.0 (29.2, 70.8)	38	36.9 (26.5, 48.7)	40	49.4 (36.6, 62.3)
	Score range	n	Median (95% CI)	n	Median (95% CI)	n	Median (95% CI)	n	Median (95% CI)	n	Median (95% CI)
Pain intensity before COVID-19	Score from 0 = no pain to 10 = worst imaginable pain	17	3.0 (3.0, 5.0)	66	5.0 (4.0, 5.0)	24	6.0 (5.0, 6.0)	103	6.0 (6.0, 7.0)	81	7.0 (7.0, 8.0)
Depression score before COVID-19	T score from 41.0 to 79.4	17	51.8 (41.0, 57.3)	65	55.7 (53.9, 57.3)	24	52.9 (49.0, 58.9)	101	58.9 (57.3, 60.5)	80	62.2 (60.5, 63.9)
Pain interference before COVID-19	T score from 41.6 to 75.6	17	55.6 (52.0, 63.8)	65	58.5 (57.1, 61.2)	24	56.4 (55.6, 59.9)	101	62.5 (61.2, 63.8)	79	65.2 (63.8, 66.6)
Physical function before COVID-19	T score from 22.9 to 56.9	17	45.3 (41.8, 56.9)	66	42.6 (41.8, 43.4)	23	43.4 (40.4, 48.0)	101	40.4 (39.1, 41.8)	79	36.7 (35.6, 39.1)
Age in years	≤50	12	70.6 (43.6, 88.2)	45	68.2 (54.4, 79.3)	15	62.5 (40.1, 80.6)	56	54.4 (42.7, 65.6)	44	54.3 (41.2, 66.8)
	>50	5	29.4 (11.8, 56.4)	21	31.8 (20.7, 45.6)	9	37.5 (19.4, 59.9)	41	39.8 (29.1, 51.6)	33	40.7 (28.7, 54.0)
	Score range	n	Median (95% CI)	n	Median (95% CI)	n	Median (95% CI)	n	Median (95% CI)	n	Median (95% CI)
Acute stress disorder	Score from 0 to 76	17	10.0 (5.0, 16.0)	66	13.0 (9.0, 15.0)	24	13.0 (8.0, 23.0)	103	13.0 (12.0, 15.0)	81	20.0 (16.0, 24.0)
Finding the COVID-19 pandemic stressful	Score from 0 (not at all) to 10 (extremely)	17	7.0 (5.0, 8.0)	66	7.0 (6.0, 8.0)	24	7.0 (5.0, 8.0)	103	7.0 (6.0, 8.0)	80	8.0 (7.0, 8.0)
Finding lockdown measures associated with the COVID-19 pandemic stressful	Score from 0 (not at all) to 10 (extremely)	17	5.0 (3.0, 7.0)	66	5.0 (5.0, 6.0)	24	6.0 (3.0, 7.0)	103	6.0 (5.0, 7.0)	81	7.0 (6.0, 8.0)
Cortisol variables											
Cortisol before COVID-19	pg/mg from 3.1 to 328.9	5	9.6 (8.4, 45.9)	19	11.4 (8.6, 19.9)	7	21.0 (6.9, 91.7)	23	9.8 (6.9, 13.5)	20	13.3 (9.3, 23.7)
Cortisol after COVID-19	pg/mg from 2.9 to 566.2	5	11.5 (10.1, 103.4)	23	14.9 (12.9, 24.4)	8	18.9 (14.4, 49.8)	29	13.6 (8.8, 23.1)	24	19.2 (14.3, 37.1)
Percentage of change in cortisol	pg/mg from −60.5 to 250.0	5	22.9 (5.0, 125.3)	19	36.2 (12.5, 96.4)	7	25.0 (−60.5, 127.5)	23	24.1 (−3.5, 50.6)	20	25.6 (−3.3, 48.5)
Time-variant variable											
COVID-19 diagnosis at any point	No	12	70.6 (43.6, 88.2)	56	84.8 (72.5, 92.2)	16	66.7 (44.0, 83.6)	95	92.2 (84.2, 96.4)	66	81.5 (70.1, 89.2)
	Yes	5	29.4 (11.8, 56.4)	10	15.2 (7.8, 27.5)	8	33.3 (16.4, 56.0)	8	7.8 (3.6, 15.8)	15	18.5 (10.8, 29.9)
Methodological variable											
Months between pre–COVID-19 and onset-of-pandemic surveys	0–17 mo	17	2.0 (2.0, 8.0)	66	6.5 (3.0, 8.0)	24	3.0 (1.0, 8.0)	102	6.5 (3.0, 8.0)	80	3.0 (2.0, 6.0)

**Table 3:** Multivariable binary logistic regression models used to identify participants' sociodemographic and clinical characteristics associated with trajectories of pain intensity (n = 230).

Variables	Categories	Stable severe and moderate vs other trajectories		U-shape vs other trajectories		Inverted U-shape vs other trajectories		Stable mild vs other trajectories	
		OR (95% CI)	*P*	OR (95% CI)	*P*	OR (95% CI)	*P*	OR (95% CI)	*P*
Pre–COVID-19 variables									
Sex at birth	Female vs male	0.7 (0.4, 1.6)	0.464	2.5 (0.7, 8.8)	0.351	1.2 (0.6, 2.6)	0.645	0.4 (0.1, 1.8)	0.251
Low back pain been an ongoing problem over the past 6 mo	At least half the days in the past 6 mo vs less than half the days in the past 6 mo	1.6 (0.7, 3.7)	0.310			0.9 (0.4, 2.0)	0.740	0.3 (0.6, 2.0)	0.228
	Every day or nearly every day in the past 6 mo vs less than half the days in the past 6 mo	**3.3 (1.4, 7.7)**	**0.007**			0.5 (0.2, 1.2)	0.131	1.0 (0.2, 4.7)	0.989
Low back pain spread down your leg(s) during the past 2 wk	Yes vs no/not sure			0.6 (0.2, 1.8)	0.391			**0.1 (0.1, 0.7)**	**0.022**
Widespread pain (pain in most of the body)	Yes vs no	**2.7 (1.4, 5.2)**	**0.004**	0.6 (0.2, 1.8)	0.369			**0.1 (0.01, 0.6)**	**0.013**
Opioid painkillers	Yes vs no					0.4 (0.1, 1.0)	0.057		
It is not really safe for a person with my low back problem to be physically active	Agree vs disagree					0.5 (0.2, 1.4)	0.200		
Feeling that my low back pain is terrible and never going to get any better	Agree vs disagree	1.8 (0.8, 3.7)	0.137						
Current smoker	Yes vs No					0.8 (0.3, 2.3)	0.692		
Body mass index ≥30	≥30 vs < 30							0.3 (0.1, 1.4)	0.112
Pain intensity before COVID-19	Score from 0 = no pain to 10 = worst imaginable pain)	**1.5 (1.3, 1.9)**	**<0.001**			**0.8 (0.7, 0.9)**	**0.014**		
Pain interference before COVID-19	T score from 41.6 to 75.6			**0.9 (0.8, 0.98)**	**0.009**				
Physical function before COVID-19	T score from 22.9 to 56.9							1.1 (0.9, 1.2)	0.278
Onset outbreak variables									
Age in years	>50 vs ≤50	0.8 (0.4, 1.7)	0.585	1.7 (0.6, 5.1)	0.143	0.9 (0.4, 1.8)	0.751	1.3 (0.3, 5.9)	0.712
Acute stress disorder	Score from 0 to 76	1.0 (1.0, 1.0)	0.656	1.0 (1.0, 1.1)	0.103	1.0 (0.9, 1.0)	0.084	0.9 (0.9, 1.0)	0.233
Finding the COVID-19 pandemic stressful	Score from 0 (not at all) to 10 (extremely)	1.0 (0.9, 1.2)	0.697	0.9 (0.7, 1.1)	0.290	1.1 (0.9, 1.3)	0.539	1.3 (0.9, 1.9)	0.110
Finding lockdown measures associated with the COVID-19 pandemic stressful	Score from 0 (not at all) to 10 (extremely)	1.1 (0.9, 1.3)	0.327	0.9 (0.7, 1.2)	0.561	1.0 (0.9, 1.1)	0.892	1.0 (0.7, 1.3)	0.814
Time-variant variable									
Having COVID-19 at any point	Yes vs No	0.5 (0.2, 1.2)	0.109	**3.9 (1.3, 11.3)**	**0.013**	0.8 (0.3, 1.9)	0.571	1.0 (0.2, 4.6)	0.965
Model evaluation									
Hosmer and Lemeshow test		0.184		0.915		0.103		0.067	

Significant associations were bolded. In the model of severe or stable moderate pain intensity trajectories, we forced age, sex, and finding the COVID-19 pandemic stressful and having COVID-19 at any point. In the model of U-shape pain intensity trajectories, we forced age, sex, acute stress disorder, finding the COVID-19 pandemic stressful, finding lockdown measures associated with the COVID-19 pandemic stressful, and having COVID-19 at any point. In the inverted U-shape pain intensity trajectory, and the slight pain intensity trajectory models, we forced age, sex, acute stress disorder, finding the COVID-19 pandemic stressful, finding lockdown measures associated with the COVID-19 pandemic stressful, and having COVID-19 at any point.

Descriptively, the trajectory of severe depressive symptoms was characterized by a high percentage of people reporting widespread pain and participants treated with opioids. Likewise, this trajectory showed a high score of depressive score before COVID-19, high levels of acute stress disorder symptoms, high levels of stress related to the pandemic and to the lockdown measures, and lower levels of physical function before COVID-19 (Table 4). In the multivariate analysis (Table 5), each additional increase of 1 point in depressive symptom score before COVID-19 (OR = 1.2; 95% CI = 1.1, 1.2), acute stress disorder (OR = 1.1; 95% CI = 1.03, 1.1), and finding lockdown measures associated with the COVID-19 pandemic stressful (OR = 1.3; 95% CI = 1.04, 1.6) were associated with an increase in the odds of having severe depressive symptoms compared with the other 3 trajectories.

**Table 4:** Sample characteristics by trajectories of depressive symptoms.

Variable	Category	Stable none		Stable very mild		Stable mild		Stable moderate		Severe slightly improving	
		n	% (95% CI)	n	% (95% CI)	n	% (95% CI)	n	% (95% CI)	n	% (95% CI)
Pre–COVID-19 variables											
Sex at birth	Female	39	67.2 (52.5, 79.2)	43	70.5 (55.3, 82.2)	63	74.1 (61.5, 83.7)	43	68.3 (53.2, 80.3)	14	58.3 (36.3, 77.5)
	Male	19	32.8 (20.8, 47.5)	17	27.9 (16.5, 43.0)	21	24.7 (15.3, 37.3)	19	30.2 (18.5, 45.2)	10	41.7 (22.5, 63.7)
Working (full time or part-time)	No	56	96.6 (86.5, 99.2)	55	90.2 (78.3, 95.9)	71	83.5 (72.7, 90.6)	53	84.1 (71.4, 91.9)	22	91.7 (70.9, 98.0)
	Yes	2	3.4 (0.8, 13.5)	6	9.8 (4.1, 21.7)	14	16.5 (9.4, 27.3)	10	15.9 (8.1, 28.6)	2	8.3 (2.0, 29.1)
Low back pain been an ongoing problem over the past 6 mo	At least half the days in the past 6 mo	13	22.4 (12.1, 37.6)	21	34.4 (21.8, 49.8)	25	29.4 (19.2, 42.2)	15	23.8 (13.5, 38.5)	11	45.8 (24.7, 68.5)
	Every day or nearly every day in the past 6 mo	22	37.9 (24.4, 53.6)	22	36.1 (23.1, 51.4)	37	43.5 (31.5, 56.4)	39	61.9 (46.9, 75.0)	12	50.0 (28.0, 72.0)
	Less than half the days in the past 6 mo	23	39.7 (25.9, 55.3)	18	29.5 (17.8, 44.7)	23	27.1 (17.2, 39.8)	9	14.3 (6.7, 27.8)	1	4.2 (0.6, 25.5)
Low back pain spread down your leg(s) during the past 2 wk	No/not sure	33	56.9 (42.4, 70.3)	27	44.3 (31.0, 58.4)	34	40.0 (29.0, 52.1)	30	47.6 (34.2, 61.4)	7	29.2 (13.5, 52.0)
	Yes	25	43.1 (29.7, 57.6)	34	55.7 (41.6, 69.0)	51	60.0 (47.9, 71.0)	33	52.4 (38.6, 65.8)	17	70.8 (48.0, 86.5)
Widespread pain (pain in most of the body)	No	35	60.3 (44.7, 74.1)	29	47.5 (33.1, 62.4)	40	47.1 (34.7, 59.8)	22	34.9 (22.4, 50.0)	3	12.5 (3.8, 34.2)
	Yes	21	36.2 (23.0, 51.9)	30	49.2 (34.6, 63.9)	44	51.8 (39.1, 64.2)	39	61.9 (46.9, 75.0)	21	87.5 (65.8, 96.2)
Opioid painkillers	No/not sure	46	79.3 (65.3, 88.6)	51	83.6 (70.5, 91.6)	63	74.1 (62.3, 83.2)	51	81.0 (67.8, 89.6)	9	37.5 (19.4, 59.9)
	Yes	12	20.7 (11.4, 34.7)	10	16.4 (8.4, 29.5)	22	25.9 (16.8, 37.7)	12	19.0 (10.4, 32.2)	15	62.5 (40.1, 80.6)
Infiltrations/injections	No/not sure	47	81.0 (67.2, 89.9)	54	88.5 (76.3, 94.9)	76	89.4 (78.8, 95.0)	57	90.5 (79.0, 96.0)	19	79.2 (54.8, 92.2)
	Yes	11	19.0 (10.1, 32.8)	7	11.5 (5.1, 23.7)	8	9.4 (4.2, 19.7)	6	9.5 (4.0, 21.0)	4	16.7 (5.5, 40.7)
It is not really safe for a person with my low back problem to be physically active	Agree	11	19.0 (10.1, 32.8)	11	18.0 (9.6, 31.3)	13	15.3 (8.5, 26.0)	12	19.0 (10.4, 32.2)	10	41.7 (22.5, 63.7)
	Disagree	47	81.0 (67.2, 89.9)	50	82.0 (68.7, 90.4)	72	84.7 (74.0, 91.5)	51	81.0 (67.8, 89.6)	14	58.3 (36.3, 77.5)
Feeling that my low back pain is terrible and never going to get any better	Agree	21	36.2 (23.7, 50.9)	23	37.7 (25.2, 52.0)	38	44.7 (33.3, 56.8)	29	46.0 (32.8, 59.9)	14	58.3 (36.3, 77.5)
	Disagree	37	63.8 (49.1, 76.3)	38	62.3 (48.0, 74.8)	47	55.3 (43.2, 66.7)	34	54.0 (40.1, 67.2)	10	41.7 (22.5, 63.7)
Excess of alcohol or drugs	No	13	22.4 (12.6, 36.6)	12	19.7 (10.3, 34.2)	42	49.4 (37.6, 61.3)	30	47.6 (34.2, 61.4)	8	33.3 (16.4, 56.0)
	Yes	45	77.6 (63.4, 87.4)	48	78.7 (64.0, 88.5)	43	50.6 (38.7, 62.4)	33	52.4 (38.6, 65.8)	16	66.7 (44.0, 83.6)
Current smoker	No	48	82.8 (68.1, 91.5)	56	91.8 (79.4, 97.0)	69	81.2 (69.2, 89.2)	42	66.7 (51.6, 79.0)	19	79.2 (56.5, 91.8)
	Yes	8	13.8 (6.2, 27.9)	4	6.6 (2.1, 18.4)	12	14.1 (7.3, 25.4)	18	28.6 (17.2, 43.5)	5	20.8 (8.2, 43.5)
Body mass index ≥30	<30	31	53.4 (38.2, 68.1)	40	65.6 (50.2, 78.2)	48	56.5 (43.6, 68.5)	35	55.6 (40.7, 69.4)	13	54.2 (32.7, 74.2)
	≥30	25	43.1 (28.9, 58.6)	20	32.8 (20.4, 48.1)	36	42.4 (30.4, 55.3)	25	39.7 (26.4, 54.7)	11	45.8 (25.8, 67.3)
Pain intensity before COVID-19	Score from 0 = no pain to 10 = worst imaginable pain)	58	6.0 (5.0, 7.0)	61	6.0 (5.0, 7.0)	85	6.0 (5.0, 7.0)	63	7.0 (6.0, 7.0)	24	7.0 (6.0, 8.0)
Depression score before COVID-19	T score from 41.0 to 79.4	58	51.8 (49.0, 53.9)	60	53.9 (51.8, 57.3)	84	58.9 (55.7, 60.5)	61	62.2 (62.2, 63.9)	24	70.3 (65.7, 73.3)
Pain interference before COVID-19	T score from 41.6 to 75.6	57	61.2 (58.5, 63.8)	61	61.2 (57.1, 63.8)	81	61.2 (59.9, 62.5)	63	62.5 (61.2, 65.2)	24	66.6 (62.5, 69.7)
Physical function before COVID	T score from 22.9 to 56.9	58	41.8 (39.1, 43.4)	60	41.8 (40.4, 45.3)	84	40.4 (39.1, 41.8)	61	41.8 (37.9, 41.8)	23	36.7 (35.6, 37.9)
Onset-of-pandemic variables											
Age in years	≤50	27	46.6 (31.9, 61.8)	34	55.7 (40.7, 69.8)	56	65.9 (52.9, 76.8)	36	57.1 (42.2, 70.8)	19	79.2 (54.8, 92.2)
	>50	30	51.7 (36.6, 66.6)	23	37.7 (24.5, 53.0)	27	31.8 (21.2, 44.7)	25	39.7 (26.4, 54.7)	4	16.7 (5.5, 40.7)
Acute stress disorder	Score from 0 to 76	58	5.0 (4.0, 7.0)	61	11.0 (8.0, 14.0)	85	14.0 (13.0, 17.0)	63	21.0 (18.0, 28.0)	24	35.5 (27.0, 56.0)
Finding the COVID-19 pandemic stressful	Score from 0 (not at all) to 10 (extremely)	58	5.0 (5.0, 6.0)	61	7.0 (5.0, 8.0)	84	7.0 (6.0, 8.0)	63	8.0 (8.0, 9.0)	24	10.0 (8.0, 10.0)
Finding lockdown measures associated with the COVID-19 pandemic stressful	Score from 0 (not at all) to 10 (extremely)	58	4.0 (3.0, 5.0)	61	5.0 (4.0, 6.0)	85	6.0 (5.0, 7.0)	63	7.0 (7.0, 8.0)	24	9.5 (8.0, 10.0)
Cortisol variables	Score range	n	Median (95% CI)	n	Median (95% CI)	n	Median (95% CI)	n	Median (95% CI)	n	Median (95% CI)
Cortisol before COVID-19	pg/mg from 3.1 to 328.9	19	9.6 (8.4, 45.9)	21	13.4 (10.0, 23.3)	19	11.4 (7.4, 45.9)	13	8.8 (5.3, 13.5)	2	17.2 (10.8, 23.7)
Cortisol after COVID-19	pg/mg from 2.9 to 566.2	25	11.5 (10.1, 103.4)	24	17.0 (14.8, 33.9)	22	14.8 (12.9, 36.2)	14	12.0 (8.4, 19.1)	4	22.9 (8.8, 161.3)
Percentage of change in cortisol	pg/mg from −60.5 to 250.0	19	22.9 (5.0, 125.3)	21	14.8 (−3.5, 41.0)	19	19.2 (12.5, 61.0)	13	45.5 (−3.6, 85.3)	2	−10.9 (−18.1, −3.7)
Time-variant variable	Category	n	% (95% CI)	n	% (95% CI)	n	% (95% CI)	n	% (95% CI)	n	% (95% CI)
Having COVID-19 at any point	No	50	86.2 (73.2, 93.5)	50	82.0 (68.7, 90.4)	69	81.2 (70.0, 88.8)	53	84.1 (71.4, 91.9)	23	95.8 (76.4, 99.4)
	Yes	8	13.8 (6.5, 26.8)	11	18.0 (9.6, 31.3)	16	18.8 (11.2, 30.0)	10	15.9 (8.1, 28.6)	1	4.2 (0.6, 23.6)
Methodological variables	Score range	n	Median (95% CI)	n	Median (95% CI)	n	Median (95% CI)	n	Median (95% CI)	n	Median (95% CI)
Months between pre–COVID-19 and baseline surveys	0–17 mo	57	7.0 (3.0, 8.0)	61	6.0 (3.0, 7.0)	84	3.0 (2.0, 7.0)	63	4.0 (2.0, 7.0)	24	5.0 (2.0, 8.0)

**Table 5:** Multivariable bivariate logistic regression model used to identify participants' sociodemographic and clinical characteristics associated with trajectories of depression.

Variables	Categories	Severe lightly improving or stable moderate trajectories vs other trajectories		Stable mild vs other trajectories		Stable very mild or stable none vs other trajectories	
		OR (95% CI)	*P*	OR (95% CI)	*P*	OR (95% CI)	*P*
Pre–COVID-19 variables							
Sex at birth	Female vs male	0.6 (0.3, 1.3)	0.204	1.4 (0.7, 2.8)	0.352	1.2 (0.6, 2.8)	0.585
Working full time or part-time	Yes vs no					0.5 (0.2, 1.5)	0.195
Low back pain been an ongoing problem over the past 6 mo	At least half the days in the past 6 mo vs less than half the days in the past 6 mo Every day or nearly every day in the past 6 mo vs less than half the days in the past 6 mo			0.8 (0.4, 1.9) 0.8 (0.4, 1.7)	0.688 0.556		
Low back pain spread down your leg(s) during the past 2 wk	Yes vs no/not sure			**2.3 (1.2, 4.4)**	**0.009**		
Infiltration/injections	Yes vs no	1.2 (0.4, 3.9)	0.784			1.9 (0.6, 5.9)	0.283
Feeling that my low back pain is terrible and never going to get any better	Agree vs disagree			0.6 (0.3, 1.4)	0.218		
Excess of alcohol or drugs	Yes vs no			**0.4 (0.2, 0.8)**	**0.010**	**3.4 (1.7, 7.0)**	**0.001**
Current smoker	Yes vs no						
Body mass index ≥30	≥30 vs < 30					0.8 (0.4, 1.7)	0.608
Pain intensity before COVID-19		0.9 (0.7, 1.1)					
Depression before COVID-19		**1.2 (1.1, 1.3)**	**<0.0001**			**0.9 (0.9, 0.9)**	**<0.001**
Onset outbreak variables							
Age in years	>50 vs ≤50	1.1 (0.5, 2.5)	0.832	0.6 (0.3, 1.3)	0.189	1.6 (0.8, 3.5)	0.199
Acute stress disorder	Score from 0 to 76	**1.1 (1.03, 1.1)**	**0.001**	1.0 (1.0, 1.0)	0.183	**0.9 (0.9, 09)**	**0.022**
Finding the COVID-19 pandemic stressful	Score from 0 (not at all) to 10 (extremely)	1.1 (0.8, 1.3)	0.665	1.0 (0.9, 1.2)	0.700	0.9 (0.8, 1.3)	0.494
Finding lockdown measures associated with the COVID-19 pandemic stressful	Score from 0 (not at all) to 10 (extremely)	**1.3 (1.05, 1.6)**	**0.017**	1.0 (0.9, 1.2)	0.858	0.9 (0.8, 1.0)	0.091
Time-variant variable							
Having COVID-19 at any point	Yes vs no	0.7 (0.3, 1.9)	0.478	1.1 (0.5, 2.5)	0.761	1.2 (0.5, 2.8)	0.709
Methodological variable							
Months between pre–COVID-19 and onset-of-pandemic surveys	0–17 mo			1.0 (0.9, 1.0)	0.287	1.0 (0.9, 1.1)	0.364
Model evaluation							
Hosmer and Lemeshow test		0.593		0.289		0.448	

Significant associations were bolded. In the model of severe or stable moderate depressive symptoms trajectories, we forced sex, having COVID-19 at any point, and finding the COVID-19 pandemic stressful. In the model of mild stable moderate depressive symptoms trajectories, we forced age, sex, having COVID-19 at any point, and finding the COVID-19 pandemic stressful. In the model of stable slight or stable nondepressive symptoms trajectories, we forced sex, age, and having COVID-19 at any point.

Of the 291 participants, 105 agreed to provide a hair sample (other participants either could not be reached by phone to explain this procedure, refused, or did not have hair long enough). Among these 105 individuals, after excluding 3 outliers (with cortisol values ≥ 3 SDs), 74 provided a sufficient hair length (6 cm).

**Figure 4.**
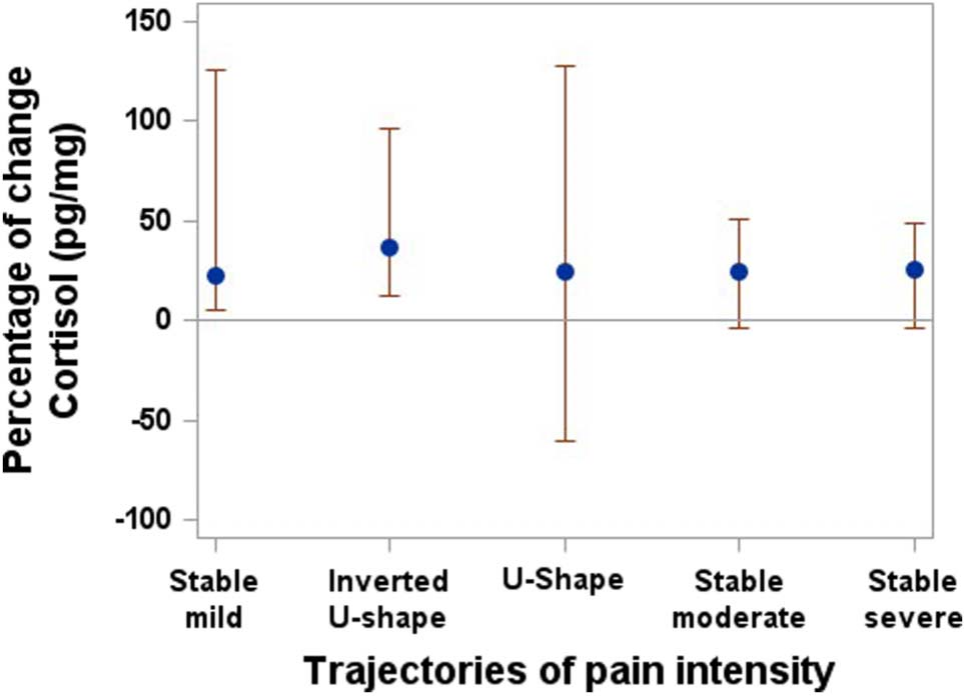
Cortisol levels by trajectories of pain intensity. Median and 95% confidence interval.

**Figure 5.**
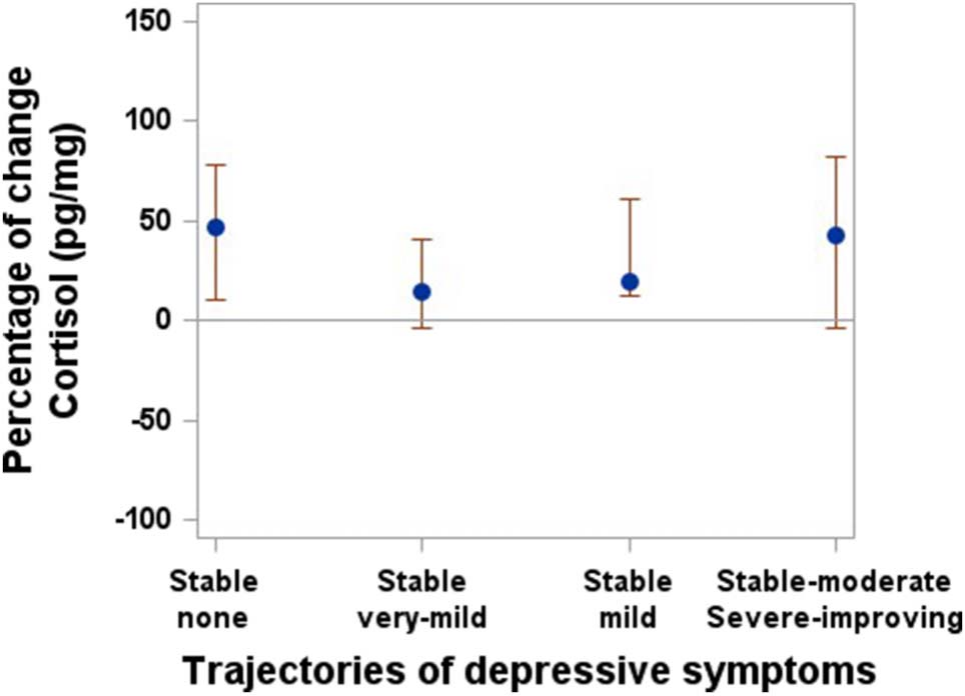
Cortisol levels by trajectories of depression. Median and 95% confidence interval.

Although the confidence intervals do not indicate a significant difference between the trajectory groups of pain intensity (Fig. 4) and depressive symptoms (Fig. 5), there was an increasing trend in cortisol levels in the post–onset of the COVID-19 pandemic measurements for the stable mild and inverted U-shape pain intensity groups, as well as for the stable none and stable mild trajectories of depressive symptoms. In the binary multiple logistic regression models that include the pre–onset of the COVID-19 pandemic to post–onset of the COVID-19 pandemic percentage of change in cortisol levels and subjective stress measures, the pre–onset of the COVID-19 pandemic to post–onset of the COVID-19 pandemic percentage of change in cortisol is not significantly associated with any trajectory of pain or depressive symptoms (Table 6).

**Table 6:** Subgroup analysis.

Variables	Categories/range	Pain intensity*						Depressive symptoms					
		Stable severe and moderate vs other trajectories (n = 67)		U-shape vs other trajectories		Inverted U-Shape vs other trajectories		Severe slightly improving or stable moderate trajectories vs other trajectories		Stable mild vs other trajectories		Stable very mild or none vs other trajectories	
		OR (95% CI)	*P*	OR (95% CI)	*P*	OR (95% CI)	*P*	OR (95% CI)	*P*	OR (95% CI)	*P*	OR (95% CI)	*P*
Cortisol variable													
Percentage of change in cortisol, pg/mg	−60.5 to 250	1.0 (1.0, 1.0)	0.547	1.0 (1.0, 1.0)	0.630	1.0 (1.0, 1.0)	0.690	1.0 (1.0, 1.0)	0.722	1.0 (1.0, 1.0)	0.753	1.0 (1.0, 1.0)	0.413
Pre–COVID-19 variables													
Pain intensity before COVID-19	Score from 0 = No pain to 10 = worst imaginable pain)	1.3 (0.8, 2.2)	0.269	1.4 (0.7, 2.7)	0.389	0.7 (0.5, 1.2)	0.202	0.8 (0.3, 1.9)	0.612	0.9 (0.5, 1.4)	0.571	1.5 (0.8, 2.9)	0.248
Depressive symptoms before COVID-19	T score from 41.0 to 79.4	1.0 (0.9, 1.1)	0.996	1.0 (0.9, 1.2)	0.553	1.0 (0.9, 1.1)	0.819	1.3 (1.0, 1.5)	0.036	1.1 (1.0, 1.2)	0.210	0.9 (0.8, 1.0)	0.035
Pain interference before COVID-19	T score from 41.6 to 75.6	1.0 (0.8, 1.1)	0.597	0.9 (0.7, 1.1)	0.244	1.0 (0.9, 1.2)	0.841	1.1 (0.8, 1.6)	0.448	1.0 (0.8, 1.2)	0.788	0.9 (0.8, 1.2)	0.612
Physical function before COVID-19	T score from 22.9 to 56.9	0.7 (0.5, 0.9)	0.007	1.1 (0.9, 1.4)	0.262	1.0 (0.8, 1.2)	0.961	1.1 (0.8, 1.5)	0.556	1.0 (0.8, 1.2)	0.716	1.1 (0.9, 1.4)	0.441
Onset-of-pandemic variables													
Age in years	Years	1.0 (0.9, 1.1)	0.730	1.0 (0.9, 1.1)	0.919	1.0 (1.0, 1.1)	0.584	1.0 (0.9, 1.1)	0.613	1.0 (0.9, 1.0)	0.544	1.1 (0.9, 1.1)	0.728
Acute stress disorder	Score from 0 to 76	1.1 (1.0, 1.2)	0.098	0.9 (0.8, 1.0)	0.192	1.0 (0.9, 1.1)	0.930	1.1 (1.0, 1.3)	0.098	1.0 (1.0, 1.1)	0.337	0.8 (0.7, 0.9)	0.004
Finding the COVID-19 pandemic stressful	Score from 0 (not at all) to 10 (extremely)	1.2 (0.9, 1.6)	0.262	1.2 (0.8, 1.9)	0.328	0.7 (0.5, 1.0)	0.038	1.7 (0.9, 3.3)	0.131	0.8 (0.6, 1.2)	0.313	1.2 (0.7, 1.9)	0.537
Finding lockdown measures associated with the COVID-19 pandemic stressful	Score from 0 (not at all) to 10 (extremely)	1.0 (0.8, 1.4)	0.677	0.9 (0.6, 1.3)	0.513	1.0 (0.8, 1.4)	0.783	1.2 (0.7, 2.0)	0.529	1.1 (0.8, 1.5)	0.403	0.8 (0.6, 1.3)	0.393
Methodological variable													
Months between pre–COVID-19 and baseline surveys	0–17 mo	1.0 (0.8, 1.2)	0.753	1.0 (0.8, 1.2)	0.716	1.1 (0.9, 1.2)	0.434	0.9 (0.8, 1.1)	0.347	1.1 (0.9, 1.2)	0.428	1.0 (0.8, 1.2)	0.809
Model evaluation													
Hosmer and Lemeshow test		0.954		0.533		0.789		0.268		0.032		0.339	

Association between cortisol and pain intensity trajectories and depressive symptoms adjusted for several variables.

* Stable mild pain (n = 5) vs other trajectories (n = 62) were not included because of small sample size.

## 4. Discussion

We investigated trajectories of pain intensity and depressive symptoms beyond the early stages of the COVID-19 pandemic in a cohort of participants living with chronic LBP. We found 5 distinct trajectories of pain intensity and 4 distinct trajectories of depressive symptoms, respectively. Pre–COVID-19 pain characteristics were associated with pain intensity trajectory groups, whereas acute stress reactions were associated with depressive symptom trajectory groups. Objective stress measure (cortisol) was not predictive of either group trajectory.

Overall, more than two-thirds of individuals experienced stable pain intensity over a two-year period, regardless of challenges or opportunities provided by the COVID-19 pandemic. This is consistent with other trajectory analyses of individuals living with LBP conducted in prepandemic states showing that <15% of cohort participants show fluctuations in pain states.^20^ It is also consistent with another study after individuals with chronic pain seeking tertiary care during the pandemic.^47^ Variability in pain states tends to be identified in studies with shorter time between measurements (eg, weekly).^20^

It is important to note, however, that subgroups of participants experienced temporary deterioration (22.7%) or improvement (8.3%) over the first months of the pandemic, which then reverted back to baseline levels. This could be associated with disruptions in daily routines and access to care. A recent study showed that for some, disruptions in accessing pharmacological (1/3 of participants) or physical/psychological interventions (2/3 of participants) in the early stages of the COVID-19 pandemic might have altered their pain states.^22^ For others, access to virtual care was improved.^4^ Contracting COVID-19 in people with pre-existing chronic pain was associated with a U-shape trajectory in our study, but did not differentiate between the other trajectories. Little available data exist on this topic; it seems, however, that COVID-19 infections are not always associated with worsened pain states; rather, biopsychosocial factors are more strongly associated with changes in pain states during the pandemic.^38^

More severe pain intensity trajectories were associated with pain frequency, widespread pain, and higher pain intensity before COVID-19. Our findings suggest that individuals with higher pain intensity levels before the pandemic were more likely to continue experiencing significant pain throughout the pandemic. Our results also suggest that individuals with pain extending beyond the low back region may be at a higher risk of experiencing more severe and persistent pain during the pandemic. Widespread pain is often associated with conditions such as fibromyalgia, which can contribute to increased pain sensitivity and a higher likelihood of chronic pain.^26^

Although between March 2020 and June 2022, there were 6 waves of COVID-19 cases,^15^ with their respective response strategies such as mobility restrictions, confinement, and vaccination,^12,16,42^ we found long-term stable depression symptoms in 4 of 5 trajectory groups during this period. This supports the idea that the local outbreak of the pandemic did not have a serious negative impact on depressive symptoms among people with LBP. It could be that our participants did not perceive the major changes of daily life during the pandemic and therefore showed stable trajectories over the course of this period. Also, it is possible that people with pre-existing mental health conditions may have had established treatment plans or support systems in place before the pandemic. This previous care could have helped them maintain stability or prevent worsening of their depression symptoms. The only previous 2-year study in people with chronic pain revealed improvements in pain intensity and depressive scores.^47^ Those finding supports evidence of resilience among patients receiving treatment for chronic conditions during the COVID-19 pandemic. However, that study did not evaluate trajectories of pain intensity or depressive symptoms.

In our study, the multivariate analysis revealed that higher scores of pre–COVID-19 depressive symptoms, acute stress disorder, and finding COVID-19 lockdown measures stressful were associated with stable moderate and severe slightly improving trajectories of depressive symptoms. These results are in line with a population-based study from Switzerland following people between July 2020 and December 2021, in which the highly affected trajectory of depressive symptoms had a greater proportion of individuals with higher levels of past depression,^35^ and acute stress disorder is a psychological state that can occur after experiencing a traumatic event, such as a life-threatening situation.^7^ The symptoms of acute stress disorder include intrusive thoughts or memories of the event, avoidance of reminders of the event, negative mood, heightened anxiety, and increased arousal. If left untreated, acute stress disorder can potentially lead to the development of post-traumatic stress disorder or other mental health conditions, including depression.^6^ The COVID-19 pandemic has brought about a range of stressors that can trigger or exacerbate mental health issues. Lockdown measures, such as social isolation, quarantine, financial uncertainties, disruptions to daily routines, and fear of contracting the virus, have had a profound impact on people's lives. These stressors can lead to feelings of helplessness, loneliness, anxiety, and depression. Experiencing acute stress disorder in response to highly stressful events associated with the pandemic, along with finding the lockdown measures stressful, can contribute to the development of severe and persistent depressive symptoms. The combination of acute stress, ongoing stressors, and social isolation can create a challenging environment for mental well-being and increase the risk of developing or worsening depressive symptoms. For this reason, engaging in self-care practices, maintaining social connections through virtual means, and seeking support from loved ones could be beneficial in coping with the challenges of the pandemic. It is essential to continue monitoring and addressing the physical and mental well-being of individuals with low back pain, as well as providing appropriate support and interventions to manage their symptoms effectively.

The finding of a nonstatistically significant, but slight increasing trend in cortisol levels post–onset of the COVID-19 pandemic compared with pre-onset of the COVID-19 pandemic levels in all groups suggests that the experience of the pandemic may have triggered a stress response within the study population. The small sample size and lack of statistical power are, however, limiting our capacity of interpretation. Still, the COVID-19 pandemic has brought about various stressors, including fears about health, economic uncertainties, social isolation, and disruptions to daily routines. These stressors can activate the body's stress response system, leading to increased cortisol production. It is important to note that a slight increasing trend in cortisol levels does not necessarily indicate pathological or clinically significant changes. It most likely reflects an adaptive physiological response to a stressful event. However, prolonged or chronically elevated cortisol levels can have negative physical and mental health consequences, including impaired immune function, increased risk of mental health disorders, and other physiological disturbances.^25^

### 4.1. Strengths and limitations

Strengths of this study include the longitudinal design with a long-term follow-up and the availability of prepandemic data. This is crucial to examine the mental health effects of the COVID-19 pandemic. However, there was a small sample size to evaluate pre–post COVID-19 differences of cortisol levels. In addition, it is important to note that our study is specific to individuals with LBP living in Quebec and may not generalize to other populations or individuals with different types of pain conditions. Nonetheless, the online recruitment allowed to recruit a diversified sample of persons living in all Quebec regions, and the QLBPS participants were found to be representative of other large random samples of adults living with chronic LBP in Canada and elsewhere in terms of proportion of women, education level, smoking, and pain intensity.^2^

## 5. Conclusions

Despite the now endemic status of the COVID-19 pandemic during which data were collected, results are important for many reasons. First, they showed that pain and depression states remained overall relatively stable for a majority of individuals, pointing to a relative resilience in this population. Results also indicate that for some, early adjustments to the pandemic negatively impacted physical and mental health status; these findings combined suggest the need to deploy targeted prevention and acute intervention strategies during major events (eg, pandemic and natural disasters) for more vulnerable individuals. Those might be individuals with higher levels of pain interference or lower levels of physical functioning.

## Disclosures

M.G.P. received honoraria from Canopy Growth and research funds from Pfizer ULC Canada for work unrelated to this study. Coauthors do not have conflicts of interest to declare.

## Appendix A. Supplemental digital content

Supplemental digital content associated with this article can be found online at http://links.lww.com/PR9/A234.
